# Using participatory and inclusive methodologies to explore inclusive education in Africa

**DOI:** 10.4102/ajod.v13i0.1486

**Published:** 2024-10-18

**Authors:** Mary Wickenden

**Affiliations:** 1 Institute of Development Studies, University of Sussex, Brighton, United Kingdom

**Keywords:** participatory, inclusive research, inclusive education, qualitative, Africa

## Abstract

**Background:**

This paper presents researchers’ experiences using participatory, inclusive research methodologies to explore aspects of inclusive education, with children with disabilities, parents, and teachers in Nigeria and Kenya.

**Objectives:**

The objective is to describe working with children and adults with disabilities, as research collaborators, alongside local INGO staff and OPD partners.

**Method:**

In Kenya we worked with 9 peer researchers with disabilities to run focus groups and interviews with children with disabilities, parents and teachers about inclusive pre-school education. In Nigeria we ran participatory workshops with children with disabilities, and their parents discussing what makes school and community settings inclusive, to inform the design of a Wellbeing and Inclusion checklist. The studies were based in pilot primary schools and Early Childhood Development and Education (ECDE or pre-school) classes in Nigeria and Kenya respectively. The data produced were recordings and notes from focus group discussions, interviews and activities and reflections from the peer researchers. Data analysis was an inclusive participatory process of thematic analysis carried out in person and online.

**Results:**

These innovative approaches demonstrate that with careful planning and support, both adults and children with disabilities can be involved very directly in research processes not just as participants but as researchers.

**Conclusion:**

We argue that using participatory, disability-inclusive approaches helps to make the findings more nuanced and genuine and the data and outputs generated uniquely grounded in people’s realities and perspectives.

**Contribution:**

These methods can potentially inform the mainstreaming of a disability inclusion approach into international development debates and activities.

## Introduction

This paper presents researchers’ experiences of using participatory, inclusive research methodologies to explore aspects of inclusive education, with children with disabilities, their parents, and teachers in two projects, in Nigeria and Kenya. The studies, which are part of the United Kingdom (UK) government aid funded (Foreign, Commonwealth and Development Office, FCDO) Disability Inclusive Development (DID) programme, are briefly described. However, the aim here is to showcase the use of participatory research methods, directly involving participants who are most impacted by the interventions being undertaken by the larger programme in which the research is nested. The studies provided in-depth insights into the participants’ perspectives on inclusion and inclusive education, in the context of two projects being run to pilot increased enrolment of children with disabilities in their local mainstream schools. The detail and results of the studies themselves are reported elsewhere. The focus here is on the use of participatory methods, their advantages and disadvantages, and the potential for more extensive use of these approaches within the disability research arena and beyond.

## Background

Underpinned by the aspirations of the United Nations (UN) Convention on the Rights of Persons with Disabilities (CRPD) (UN 2006) and the Sustainable Development Goals (SDGs) agenda (UN 2015, 2018), the most prominent rhetoric within the disability sector currently is the mantra ‘nothing about us without us’. This is understood to refer to policy formation, inclusive interventions and service provision across various sectors (e.g. employment, health, education, community development) and demands regular consultations and engagements at community level about a range of issues affecting disabled people (Charlton 1998; Nind 2014). A move towards disability inclusion has been promoted by various UN bodies and lobbying groups (Wescott, MacLachlan & Mannan 2021; UNDESA 2013, n.d.). However, importantly but less recognised perhaps, this disability aware and inclusive approach should also apply to research activities. Explorations and investigations about the lives of disabled people^1^ should necessarily involve the target population directly in the research processes as far as possible. This is arguably a less straightforward arena for truly participatory and inclusive approaches than other activities. Career researchers who have spent years honing their specialist skills may be unaware of the need or be reluctant and might inadvertently put barriers in the way of disabled participants’ involvement in research processes.

### Participatory research

Participatory research has become a popular choice of investigative approach in many community development and international development arenas in the last three or four decades (Bergold & Thomas 2012). There are a number of variations in exact philosophy and methods, and in the names and acronyms used, for example, Participatory Rural Appraisal (PRA) (eds. Reason & Bradbury 2008), Participatory Action Research (PAR), Community Based Participatory Research (CBPR) (Greenwood 2016), and many others (Burns, Howard & Ospina 2021). There are of course subtle differences in core concepts between them, but essentially in common to all is a commitment towards engaging meaningfully with the study population of interest and involving them in many if not all aspects of the research process, including: conceptualising the research idea and questions, advisory roles, choice of methods and planning, generating and gathering data, analysis and interpretation and dissemination of findings. The extent to which participants are involved in all of these is hugely variable, but a spirit of equity, openness and involvement is vital. Co-production, the joint generation of the findings, is seen as foundational (Barke, Thomas-Hughes & Howard 2020; Thomas-Hughes & McDermont 2021). An underlying assumption is that the members of the community are ‘experts’ in their lives and their perspectives are key to understanding the topic of interest, whatever that might be (eds. Kindon, Pain & Kesby 2007; Ospina 2021). Research is seen as a relational activity, where the relationships between the researcher and the participants are equalised as far as possible, power gradients are flattened; therefore, there is more equality between different people than is perhaps usual in most research contexts (Chambers 1997; Gaventa & Cornwall 2008). It is a dialogic endeavour, involving mutual enquiry and learning for all. Therefore, in participatory research ‘the other’ is a co-producer of the new knowledge. There is a strong influence from the work of Freire, where a process of conscientisation, a ‘coming into awareness’ is key. Participants, through being involved, gain deeper insights into their own life worlds and situations. They are developing local knowledge through reflection and participation, not just increased awareness, and this may lead to subsequent actions and change.

Thus, the usual dominant research epistemologies, which assume the dominance and validity of certain types of (often positivist, objective research-driven) knowledge, are challenged, and the understandings and experiences of people and communities themselves are sought, privileged and valued in the interests of driving social transformation from the ‘bottom up’ (Gaventa 2006). Often a wide range of creative, visual and performative methods are used in participatory research, as well as more flexible and discursive verbal approaches such as storytelling and narrative enquiry (Lewin & Shaw 2021; Lewis & Hildebrandt 2020). These then provide maximum opportunities for participation, including for people who may find purely verbal (spoken or written) formats intimidating or impossible.

### Disability inclusive research

Arguably, all disability aware research should necessarily be inclusive in its ethos and practice, and intentional in its design, with the aim of ensuring that people with a range of impairments and access and/or support needs can participate as much as anyone else. The provision of whatever support and accessibility adaptations are needed is foundational to disability inclusive research as it tells potential participants that their contribution will be listened to and taken seriously. Debates have raged over the last 40 years or so about the status of and rules of engagement around doing research with disabled people and about disability (Oliver 1992; Zarb 1992). The relationship between researchers and those they are researching, is a sensitive issue and often contested. This includes discussion about the role of non-disabled researchers (Stone & Priestley 1996), about what makes methods inclusive (Kitchin 2000) and whether all research should necessarily be framed as emancipatory (Barnes 2003; Berghs 2017).

There has been an acceleration in the development and use of disability inclusive participatory methodologies since the launch of the groundbreaking UN CRPD in 2006 (UN 2006). This treaty underlines the rights of disabled people to equal citizenship and specifies their right to participation in any affairs that relate to them, epitomised in the mantra ‘nothing about us without us’ (Charlton 1998; see UNCRPD articles 4.3, 32 and general comment 7). Thus, the process of conscientisation aforementioned is coming into action, through the gathering and amplifying of disabled people’s voices, talking about a wide range of issues. The opportunity for these counter-narratives to challenge dominant ablest, stigmatising and exclusionary discourses about disability and being different are being taken up and sometimes people’s perspectives are being sought for the first time. This trend has been driven in large part by the very active global network of Organisations of People with Disabilities (OPD), who have become increasingly well organised and vocal (e.g., IDA 2022). People who have habitually been internally oppressed are now being asked for their views and expressing them strongly, given the opportunity and through the use of inclusive methods (Reeve 2014; Shevlin & Rose 2022).

Working with people with disabilities as active participants in research and increasingly as co-investigators as part of research teams, is producing more authentic data, giving insights into their worlds and concerns, and during the analysis of their interpretation of situations. Interest in and respect for their views is growing, and professional researchers are realising that without these insider views, their findings will sound hollow and will lack nuance. Disabled people’s active involvement in research is becoming seen as essential. Co-productions, powerful and inductive processes that collect and analyse data from the bottom-up, are becoming recognised for their value. The extent to which disabled people are becoming involved in all stages of the research process is variable, as is their level of participation and type of engagement. Thus, an aspiration would be that they will contribute to all stages: research design, data generation, analysis and interpretation, validation and dissemination. However, this is rarely the case as yet. There is a small but growing number of trained ‘career’ researchers who identify as disabled, and this is increasing as access to education is improving globally, and therefore this is a career choice that has become possible.

Disabled people are now getting involved in a variety of participatory inclusive research and engagement activities, although this is still more common in high than middle or low-income countries (Kuper et al. 2021). Often these events are mediated through OPD, although this should not be the only route to recruiting participants or co-researchers, as many disabled people are not members of these organisations and non-members’ views should not be excluded. Additionally, there is still a tendency for people from the more stigmatised impairment groups (e.g. those with communication, cognitive, psychosocial and complex difficulties) to continue not to be invited into research spaces. There is even now some way to go before truly equitable participation is achieved (Wickenden 2023b, Wickenden & Lopez Franco 2021; Shaw & Wickenden 2022). Several of the international disability focussed non-governmental organisations (NGOs) have produced useful resources about inclusive practice (CBM 2012; Light for the World 2017), although these are not focussed specifically on research.

Gradually, literature about including people from specific impairment groups is emerging, which is a positive sign. For example, there are studies about research relationships with people with learning disabilities (Johnson & Walmsley 2003; Kahonde 2023; Nind & Vinha 2014; Walmsley 2001) and about tackling stigma directed at this group (McConkey, Kahonde & McKenzie 2016). Some authors have written about the successes and challenges of doing research with people who are blind or who have multisensory impairments (Jaiswal et al. 2018; Watharow & Wayland 2022).

The underlying motivation for the two studies described next was to be both participatory and disability inclusive in the research approach.

## Research methods and design

### Background to the two studies

The two studies described were both research projects run in parallel and collaboration with multiple international non-governmental organisations (INGO) partners (Sightsavers, Humanity and Inclusion (HI), Leonard Cheshire and SENSE International), carrying out intervention activities in relation to developing successful models for the roll out of inclusive education in Kenya and Nigeria. The two separate projects were part of a larger overarching programme funded by the UK government (FCDO) and the DID programme. This comprises a consortium of INGOs, research entities and OPD, working together over six years (2018–2024), mainly in five countries in Africa and South Asia. The projects within this programme have focussed on trialling innovations in four different thematic areas (education, livelihoods, health and tackling negative stereotyping). However, the majority of the projects have focussed on aspects of inclusive education as this was a theme prioritised by partners within the countries (see Inclusive Futures, https://inclusivefutures.org).

### Ethical considerations

Ethics protocols for both studies described here were submitted to the Institute of Development Studies (IDS) Ethics Committee (Project no. PT/17012) and also in collaboration with our partners in Kenya and Nigeria to in-country Ethics Review Boards (ERBs) as appropriate. In both cases, particular attention was given to the extra risks, actions and factors that need to be considered when doing research with people with disabilities, as well as with children with disabilities who were involved as participants in both studies. The research team had a strong awareness of the specific risks and mitigations that might arise in relation to participatory, inclusive research and co-production processes (Barke et al. 2020; Carey & Griffiths 2017). We see it as important to elucidate these points in ethics applications for two reasons: (1) to educate ethics boards as to the risks and benefits of including disabled people and disability issues in research, and (2) to ensure that the research team, consultants, peer researchers and project partners involved would be well informed and prepared to deal with any problematic aspects which might arise. Our experience is that not detailing these aspects explicitly, can lead to ERBs not approving disability related research for what might be regarded as the wrong reasons (e.g. overprotection, lack of recognition of disabled people’s agency and right to be heard, assuming that proxies’ views are good enough, etc).

## Results

### Example 1 – Exploring perceptions of inclusion, as part of promoting disability inclusive early child development and education (ECDE) in two counties in Kenya

The intervention project in Kenya took place in the counties of Homa Bay in the West of the country, a fertile area on Lake Victoria and Kakuma refugee camp in Turkhana county in the arid north. The implementing INGOs worked with a total of nine selected pilot mainstream primary schools to support them to enrol and support children with disabilities into their pre-school (ECDE) classes. A range of different interventions were rolled out, including training for the teachers and parents, awareness-raising and advocacy in the community, and work with Ministry of Education staff and other educationalists nationally and locally. A quantitative study about children’s educational progress compared with control cohorts was also conducted.

The qualitative research study described here aimed to explore in depth the experiences and perceptions of three types of participants (children with disabilities, their parents, and teachers). It explored their ideas about inclusive pre-school education at school and also aspects of inclusion in the community. (For detailed descriptions of the study, see Wickenden, Njungi & Rohwerder 2023a, 2023b; Wickenden, Rohwerder & Njungi 2022.)

### Peer researchers as part of the team

An innovative aspect of the study was working with nine peer researchers with disabilities recruited locally in collaboration with OPD (six in Homa Bay, three in Kakuma). They were five women and four men, and they had a mix of impairments (physical or visual impairment, and one was a parent of a disabled child). Applicants were encouraged to apply by local OPD, irrespective of impairment type; thus, this characteristic was not one of our criteria. They had a mixture of education levels and previous research experience, and were selected on a range of criteria including: knowledge and experience of activism about disability and inclusion and language skills. It was important to recruit a team who could between them speak all the local languages that might be needed as well as English. They were formally contracted and paid for their work on a daily rate as advised by the INGO team, as well as receiving various travel and subsistence allowances.

They had online and face-to-face training in participatory research theory and practice. With the support of a Kenyan research consultant and the UK team, the peer researchers then worked in small groups to run separate focus groups with children with disabilities, parents and teachers involved in the initiative. Interviews were also undertaken with parents of children with more severe impairments who were on a home support programme run by Sense International. The focus groups were held in the schools, and the interviews in family homes. The types of impairments that the children had were not a criteria for selection and were not under the researchers’ control, as it depended on which children had been admitted to pre-school classes and were available to participate on the day.

### Research process

There were two rounds of data collection, first near the start of the intervention, before many of the INGO-led project training and awareness activities had started and then about 15 months later near the end of these activities (2021–2023). The research was undertaken during the coronavirus disease 2019 (COVID-19) pandemic; therefore, Kenyan government guidelines and restrictions were adhered to. The recruitment of participants, briefing of schools and logistical arrangements were facilitated by the locally based staff working for INGOs and by OPDs.

The data collected were a combination of recordings of discussions, notes from the focus groups and interviews, visual materials, such as mind maps and drawings generated during the sessions and reflections from the researchers’ debriefs after each data collection event. These were written up by the consultant. The peer researchers’ personal reflections on the experience of being a researcher were also collected during online and face-to-face debrief sessions (written and video). They were also involved in reviewing and commenting on the various reports and published papers produced. They gave permission for their videos to be shown during live presentations and webinars.

A participatory process was used to involve the whole team in thematic analysis of the data. Round one was undertaken online with the Kenyan consultant in person with the peer researchers. For the second round, the process was in person at the two sites with support online from the UK team. Key themes were identified and were mapped and clustered. Discussion generated some clear patterns across the two sites, with many noticeable similarities as well as some differences between them and across the two time points. A second round of detailed analysis involved uploading all the material to NVivo. The UK team and Kenyan consultant then did a further thematic analysis to nuance the participatory analysis process and identify relevant quotes to provide supporting evidence.

The children, parents and teachers all responded positively to the focus groups and interviews, and enjoyed having a chance to express their views and feelings. It was particularly noticeable that they were more forthcoming and talkative during the second round of data collection, when they were familiar with the approach, knew the peer researchers and had experienced more interventions, such as being in school and receiving training among others.

The children, parents and teachers were not involved in analysis and publications processes. Organisations of People with Disabilities steering group committee members were involved in reflection on the key findings during their regular and final project meetings and other dissemination events.

As this qualitative research was nested within a bigger project, the participants had the opportunity to be involved in various meetings at different time points and at the close. There was extensive discussion about next steps between local and national stakeholders, with the INGOs running the interventions and follow on work is currently being considered.

### Findings about the methods used

Overall, there was agreement among the parents and teachers that having the peer researchers facilitating the discussions and interviews was a good idea. It was inspirational for the participants to see that disabled people could work as researchers and it provided them with encouragement that disabled children could also aspire to such roles in the future. It therefore underlined the importance of access to education for the children. They did however also discuss some ways in which the education and support of disabled children could be improved.

The peer researchers were very positive about the experience of working as researchers. Their key points of learning were:

- had learnt skills which would be useful to them in the future: communication, qualitative research (including being inclusive, collecting data, analysis), organisational, teamwork, advocacy
- were more confident and informed about disability and inclusion
- greater understanding of families’ situations and of services available in the area
- increased sense of being activists and role models.

### Example 2 – Developing an inclusion checklist with disabled children and their parents as part of an inclusive primary education pilot in Kaduna State, Nigeria

The intervention project in Nigeria was called SMILE (Support Mainstreaming Inclusion so all Learn Equally) and was led by INGO Sightsavers along with a steering committee of OPD and other experts in inclusive education in Nigeria. Six mainstream primary schools in Kaduna state were supported with a variety of interventions to develop community awareness of inclusion, the schools’ inclusive practice and increase the number of children with disabilities enrolled. The project carried out a range of activities including: teacher and parent training, community advocacy, inclusive children’s clubs, work with national and state level educationalists, teacher trainers and Ministry of Education.

The associated research project reported here aimed to use work alongside the intervention activities, using participatory inclusive methods to develop an accessible checklist in collaboration with some disabled children now attending school and their parents. This would ask children with disabilities themselves and their parents about their experiences of wellbeing and inclusion in school and at home. A local research team of two consultants with experience of disability or childhood research was recruited, along with two members of the SMILE steering committee (people with lived experience of disability) who acted as advisors. This team was given online training in the concepts of inclusion and disability, inclusive and participatory research and working with children.

Three-day participatory workshops were held separately for two groups of children with disabilities and one day sessions for their parents. The venues were two of the pilot schools, familiar settings for the children. Through using a range of play based activities, trust was built between facilitators and participants. It was important to establish that this was different from school classes, there were no right or wrong answers, and children of different ages, genders and impairments worked together.

After icebreakers and ‘getting to know each other’ games, and establishing some group rules, a series of fun activities were devised to investigate their views on what impacts wellbeing and inclusion at school and also in the community. Care was taken to make sure these activities were inclusive of all and did not require literacy or verbal skills necessarily. Creative and arts-based methods were used extensively. For example, a ball game as an introduction and a large flipchart sketch of a school and of a village were placed on the floor. The children then drew or wrote or put stickers on to show what was important in each place (e.g., the classrooms, the toilets, the village pond). They also indicated places they liked or didn’t like, and discussed why (using happy or sad stickers). Everyone was given the time and space to express their views with the support of the team. Topics arising from these discussions then became question items in the draft Wellbeing and Inclusion Checklist.

In the parents’ workshops, they discussed what inclusion of their children meant to them. They were asked to think about what questions they would ask another parent about their children’s school and home life. They generated a list and then role-played asking the questions to each other.

The data from the workshops was thematically analysed and discussed in online whole team meetings. A list of 10 questions for children and 10 for parents was compiled, and a checklist format using emoticon faces on a 5-point Likert scale was developed.

The draft checklists were piloted in the same two schools but with different children and parents to evaluate how well they worked in assessing subjective experiences of wellbeing and inclusion. This was repeated after a year with revised version of the checklists, with the aim of revealing any change in the experiences of the children, given that various interventions had taken place in the meantime. Views were sought from participants about the checklist after they had participated. The revised version was felt to work more successfully than the first one. Changes included adding some extra questions, rewording some and adjusting the layout to make more space for respondents’ verbatim comments. However, further potential improvements were also identified. (For detailed description of the checklist development, suggested refinements and possible further developments, see Wickenden, Thompson et al. 2023a, 2023b).

The feedback received from the children and the parents was that they enjoyed the design workshops, developing and completing the checklists. Some parents reported that they had not had the opportunity to think and talk about their children’s school and home life before, and they appreciated this.

The two consultants who had not worked on a project that was as participatory and inclusive as this one, reflected positively on the experience. They had initially been sceptical that it would be possible to engage the children in discussion about inclusion. They were not sure that they would be able to generate questions or respond to them. Similarly, they were surprised that the parents, once they understood the task, responded very actively and were keen to contribute to designing the checklist for other parents.

## Discussion

### Benefits of participatory inclusive approaches

Overall then, our experience of using participatory and inclusive approaches with three groups—children with disabilities, their parents and teachers—demonstrates that all can be asked for their opinions, if this is done in a disability aware way with appropriate inclusive methods and adaptations. Additionally, both children and adults with disabilities can be involved in research as collaborators and peer researchers if they are given appropriate training and support.

Making sure that the activities and methods used enabled the participation of all, albeit in different ways at different levels of complexity, is a fundamental principle. Using ‘multi-modal’ methods was key (always using more than one communication method or mode, e.g., spoken words were accompanied by pictures, symbols or signs), children’s discussion was always accompanied by something for them to physically do or look at (e.g. using objects and pictures). There was a carefully planned mix of types of tasks; therefore, some were physically active and others less so. Appropriate levels of help and support are also important, as disabled people (including children) often have specific views about how they are supported. They say that if they are not helped enough this excludes them, but if they are helped too much it is patronising and denies them agency. Getting levels and types of support right is therefore absolutely essential to disability inclusive research.

Capturing the first-person perspectives of people with disabilities (whether children, adults or families) provides ‘real’ grounded data and allows subjective experiences to be revealed and recognised as important. This is humanising, demonstrating that a group of people who may be stigmatised and regarded as of reduced worth, have opinions on many topics just as others do. Asking children themselves (even very young children) is also important, because they may have views that are different from proxies such as their parents, who are more often asked about their lives. Parents may be surprised at what their own children say!

Doing research about disability in a participatory way underlines the relational nature of disability, demonstrating that a group of people often regarded as ‘the other’ can be related to in ordinary ways. Some of the data that emerged in these two studies showed that the concerns of the children, parents and teachers were in many ways similar to those of others. For example, the children were worried about bullying and about lack of resources at school (lack of books, dirty toilets, which arguably non-disabled children might also mention), the parents had worries about safety, household finances and the future for their child. The teachers were concerned about needing more resources in school and more training, heavy workloads with many children in their classes and their own careers.

Thus, findings from disability focussed research can do three things. Firstly, reveal disability-specific aspects that are different from others’ views and have not previously been known. Secondly, it can demonstrate the ways in which people with disabilities’ lives and concerns are similar to everyone else’s and this is important in relation to people being accepted and understood. Thirdly, it can illustrate the impact of endemic structural violence in communities and systems, when people tell stories of disadvantage and discrimination. There is then a possibility of righting epistemological injustice, as excluded groups’ perspectives are now recognised and their particular knowledge and experience becomes valued (Danermark & Coniavitis Gellerstedt 2004; Fricker 2007). In addition, not only will others understand disabled people better, but if, as was demonstrated with the peer researchers in Kenya, a process of conscientisation takes place. They have then gained awareness, knowledge and insights which can lead to them developing increased advocacy skills and agency.

This research was embedded in the local contexts and informed by in-country colleagues in Kenya and Nigeria. The building of relationships with participants and the data collection were led by Kenyans and Nigerians, respectively. The topic guides and activities were designed using a co-production approach, with many suggestions and adaptations being made by the consultants, peer researchers and OPD advisors on both projects. Although the funding and academic research leads were external (and outside Africa), from ex-colonial contexts, effort and care were taken to ensure that the design, data collection, analysis and dissemination were as locally crafted and influenced as possible. However, it should be acknowledged that a power gradient between the overseas-funded academic researchers and the in-country consultants, partners and participants inevitably existed. The team tried hard to flatten this disparity, by modelling respect, affirmation and appreciation of everyone’s contributions.

### Dilemmas and difficulties

Despite all the positive reasons mentioned above to do more participatory and inclusive qualitative research, there are some risks and cautionary tales to tell. There is, for example, always a risk of tokenism. Inclusive practice that is not done with enough sensitivity, responsiveness to the needs of the participants, and enough resources can be as harmful as any other type of exclusion. It is important to make sure that the whole research team are well trained, sensitised and prepared to avoid this. Asking someone what kind of support they need and then not providing it sufficiently is likely to cause frustration, mirroring previous exclusionary experiences and potentially perpetuating people’s feelings of being oppressed, abused and not being taken seriously.

Additionally, it is important when recruiting participants and introducing the project aims, to take care about how inclusion criteria are explained. It is possible to reify (bring into being) an identity that someone does not recognise for themselves, which may be upsetting or harmful. For example, young children with disabilities may not have identified themselves as different from their peers in this way (even if they have a visible impairment such as a mobility, vision or hearing difficulties). Older children and adults may choose not to identify as disabled, although they have impairments. Thus, the way that they are addressed when invited to join in could be hurtful and a revelation to them if not worded carefully. The approach used needs to be agreed among the team, taking into account local language and understandings, and the children’s previous experiences.

A question remains about how people from the most marginalised impairment groups can be included more? It is common to see disability focussed research being done that does not attempt to include those at the bottom of a hierarchy of impairment. People with cognitive, communication and psychosocial difficulties are still to a large extent excluded, stigmatised and not engaged with (Inclusion International 2006, n.d.). They are seen as difficult or expensive to include. This can be true even within disability rights focussed activities (Allport 1954; Deal 2003). There needs to be a concerted and intentional effort to develop the skills and confidence of researchers, INGOs and OPD, in including these more marginalised groups, both in interventions and in research (Shaw & Wickenden 2022). Otherwise, their perspectives will continue to be unheard and their exclusion will continue (Inclusion International 2006, n.d.).

Another dilemma is how disability can be more recognised as an aspect of identity, and therefore included in intersectionality debates and considerations. Discussions about intersecting identities now abound, but still have a tendency to focus on race, gender and sexuality. Disability as an identity is very often left out of the picture, despite people with disabilities commonly emphasising that their other identities are often as important as their disabled identity if not more and yet the combination (e.g. of being a woman and disabled) is crucial to understanding their situation and needs (Wickenden 2023a).

Finally, there remains the wicked question of how we can achieve the inclusion of a disability lens in all research, that is, not just on disability focussed topics? Ideally, all ‘mainstream’ research on any topic should by default be designed to include disabled people in its recruitment, whether qualitative or quantitative studies. We should see that the 16% of the global population who are disabled (WHO 2022) are proportionately recruited in any study populations, across the full range of sectors and areas of investigation. This would then be a major contribution to the aim of ‘mainstreaming disability into international development’.

## Conclusion

The unique aspect of this participatory disability inclusive approach is working with people with disabilities themselves, both adults and children, as participants contributing data about themselves, as well as research collaborators, alongside local employed INGO teams and OPD partners and in co-production processes as far as possible. This was somewhat curtailed by the timing of the work being during the COVID-19 pandemic.

In both Kenya and Nigeria, we used a range of innovative, inclusive methods to gather data with children and adults. The activities were designed to ensure that everyone, whatever their impairments, ages, access needs or levels of education could join in and contribute their perspectives and experiences on the subject of inclusive education and wellbeing. It is significant that young children with disabilities participated in research about their lives. The studies show that including them is both possible and important to do. Additionally, parents and teachers enjoyed being asked for and expressing their views. The team of peer researchers with disabilities working on the project in Kenya felt more knowledgeable and confident as a result of being involved in the research. They had an increased sense that they were role models for other disabled people in their community and that their skills and status had been increased.

Research that is done in this participatory and inclusive way provides data that adds depth and nuance, and can potentially complement quantitative data in mixed methods studies. It sets out to answer why and how questions rather than just focussing on what works and what doesn’t. These approaches and methods have the practical potential to inform and could lead to interventions that are suggested and validated by the population in question. This is potentially empowering and emancipatory.

## Footnotes

1 I am deliberately using this language interchangeably with ‘people with’ language with no particular connotation either way. Ref Disability Rights UK, Social Model of Disability: Language.
